# Combined Klippel-Feil syndrome, Sprengel deformity, and diffuse large B-cell lymphoma: A rare case report

**DOI:** 10.1016/j.radcr.2024.12.026

**Published:** 2025-01-10

**Authors:** Golnaz Lotfian, Amirmasoud Negarestani, Sarah Gondek, Aleksandr Raskind, William Chen, Emad Allam

**Affiliations:** Loyola University Medical Center and Loyola University Chicago, 2160 S First Ave, Maywood, IL 60153, USA

**Keywords:** Klippel-Feil syndrome, Sprengel deformity, Omovertebral bone, Lymphoma, Cervical spine

## Abstract

Klippel-Feil syndrome (KFS) is a rare congenital disorder characterized by the fusion of cervical vertebrae, with a clinical presentation that can vary widely due to genetic and phenotypic diversity. While KFS can occur as an isolated anomaly, it is often associated with other congenital conditions, such as Sprengel deformity, which may present with or without an omovertebral bone, complicating diagnosis and management. This particular case also involves diffuse large B-cell lymphoma (DLBCL), the most common subtype of non-Hodgkin lymphoma. We hereby present a complex case of a 60-year-old male with the co-occurrence of KFS, Sprengel deformity, and DLBCL. Diagnostic imaging revealed an ill-defined right neck mass on ultrasound which was confirmed on neck CT. The CT also demonstrated an elevated left scapula and a left omovertebral bone, indicative of Sprengel deformity. There was fusion of the C5 and C6 vertebrae consistent with KFS. A whole-body F-18 FDG PET scan demonstrated significant uptake in the neck mass, leading to a biopsy that confirmed DLBCL. This case highlights the importance of comprehensive medical and imaging evaluations in diagnosing and managing the complexities associated with these disorders. In particular, awareness of the potential co-existence of congenital abnormalities and aggressive malignancies is critical in such cases.

## Background

Klippel-Feil syndrome (KFS) is a rare condition where one or more cervical vertebrae are fused [1]. The inheritance pattern of KFS may be autosomal dominant, autosomal recessive, or sporadic [2,3]. KFS can be associated with other congenital conditions such as Sprengel deformity, urinary malformations, scoliosis, hearing issues, and neurological problems [4,5]. Signs and symptoms include a low hairline, neck and back pain, reduced mobility, and respiratory difficulties.

A Sprengel deformity is a rare congenital condition where the scapula is abnormally high due to issues during embryonic development [6]. This condition leads to cosmetic deformities and limited shoulder mobility. It is often associated with Klippel-Feil syndrome. The Sprengel deformity is classified into 4 grades, and treatment is often dependent on the severity of the symptoms. A subset of patients with Sprengel deformity have an abnormal osseous and/or fibrous connection between the cervical spine and scapula known as an omovertebral bar that is often associated with additional symptoms and decreased mobility [8].

Here we describe a rare case of an adult with combined KFS, Sprengel deformity with an omovertebral bone, and congenital unilateral renal agenesis, who was also diagnosed with diffuse large B-cell lymphoma. No association between KFS/Sprengel deformity and DLBCL has been previously documented; this case highlights an exceptional occurrence of these conditions. Awareness of such associations can enhance diagnostic accuracy and guide management strategies in similar clinical scenarios.

## Case presentation

**Fig. 1:**
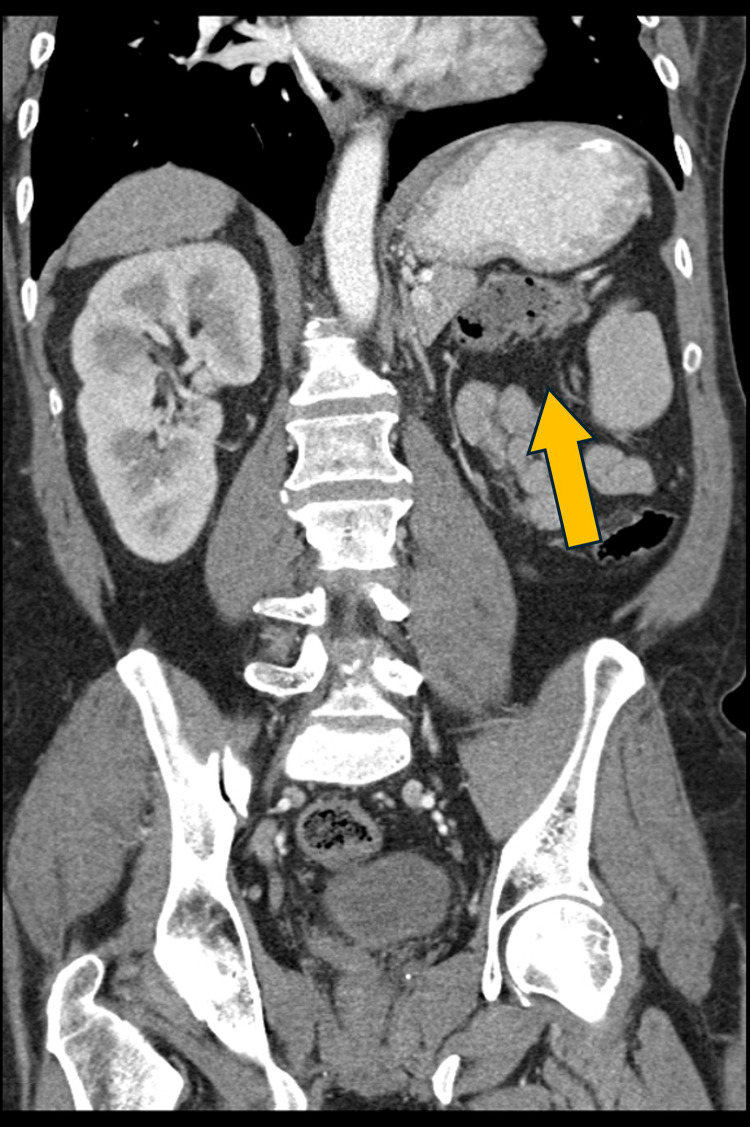
CT shows unilateral renal agenesis (yellow arrow) in this patient, which has been documented as a rare occurrence associated with KFS.

A 60-year-old male presented for evaluation of a right neck mass. His medical history included alcoholic liver cirrhosis, managed with TIPS, and congenital absence of the left kidney (Fig. 1). Initial assessment of the right neck mass with ultrasound revealed a heterogeneous mass characterized by varying echogenicity and internal vascularity, suspicious for malignancy.

Subsequently, a neck CT scan was performed, which confirmed the presence of the right neck mass, measuring approximately 7.5 × 4.4 × 5.2 cm. The CT revealed that the mass was ill-defined and in close proximity to the right thyroid lobe, although it remained unclear whether it originated from the thyroid tissue itself.

**Fig. 2:**
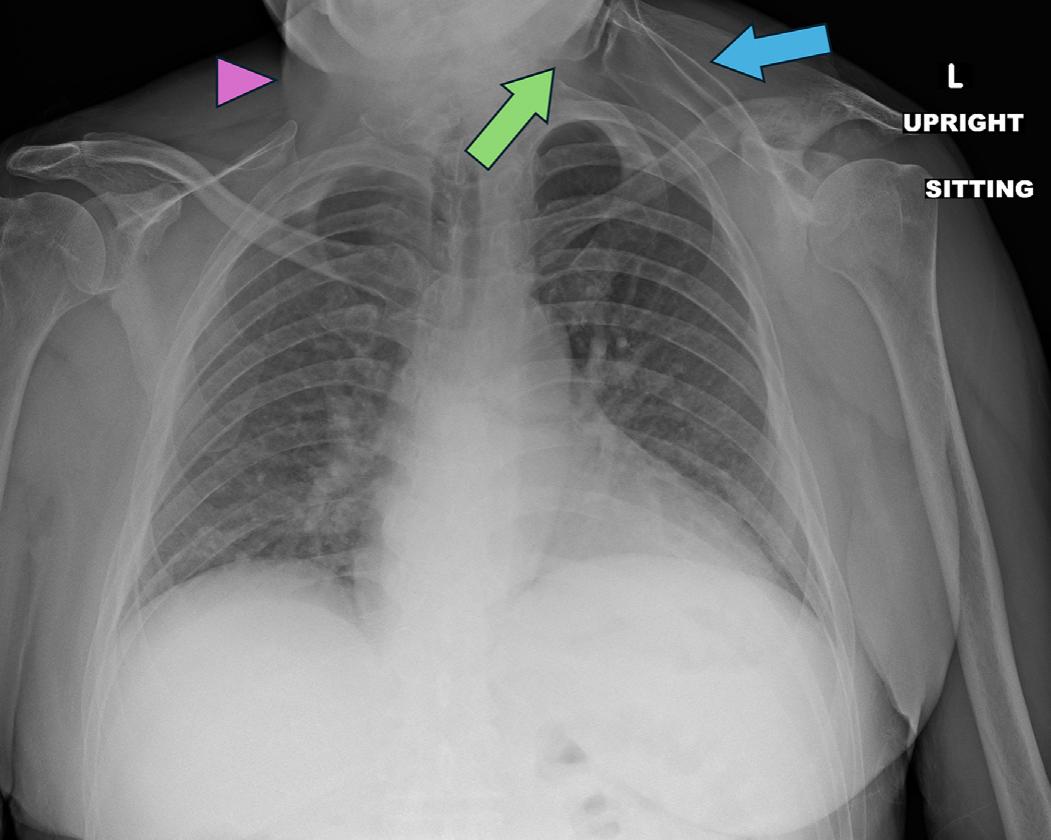
Frontal chest radiograph demonstrates a left omovertebral bone (green arrow) with scapular pseudo-articulation and left scapular elevation (blue arrow). There is suggestion of a right neck mass (pink arrowhead).

**Fig. 3:**
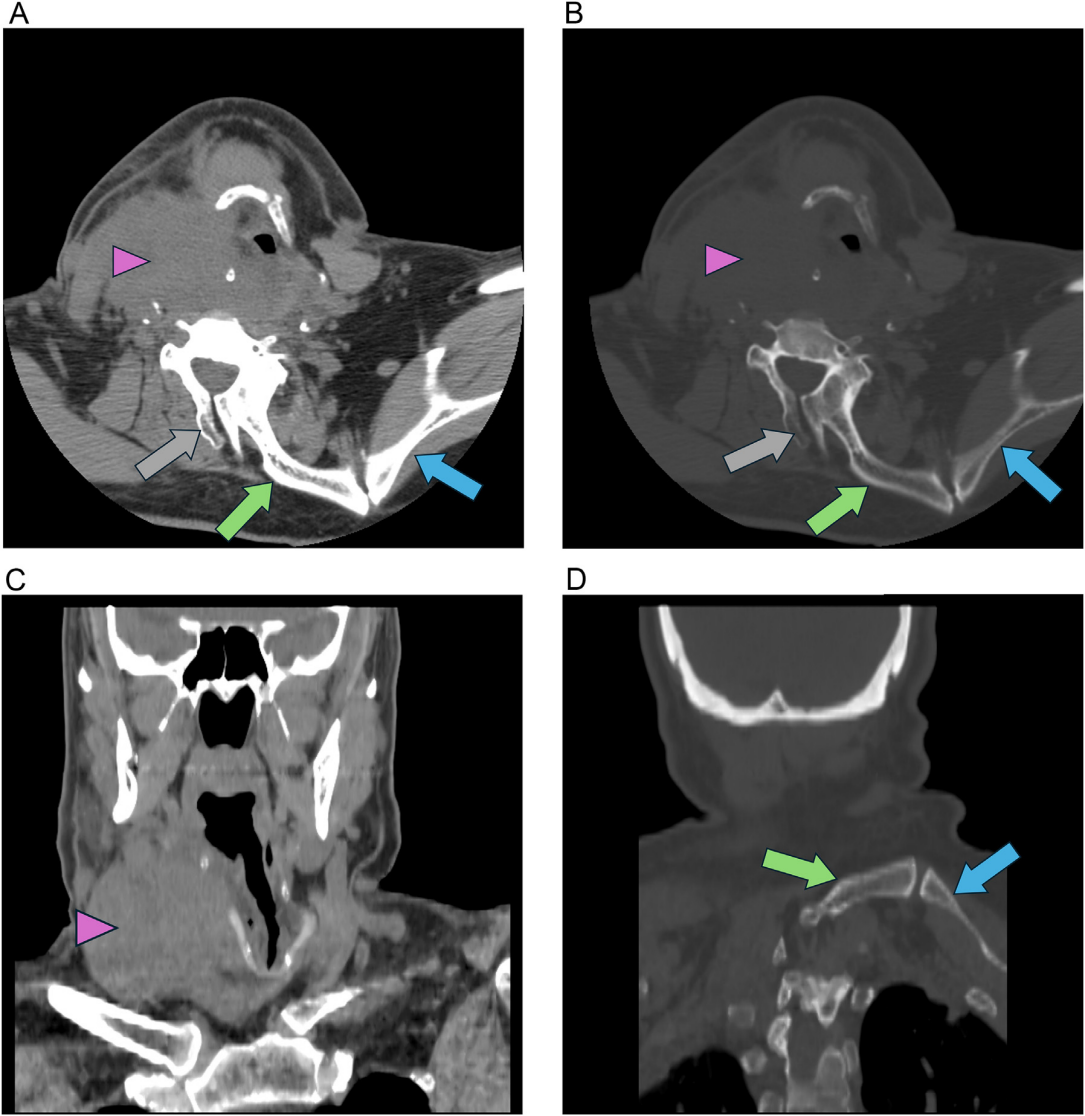
(A-D) CT of the neck shows a right neck mass (pink arrowhead) with leftward tracheal deviation. There is an abnormal bony structure extending from the C6 posterior elements to the left scapula with pseudo-articulation, consistent with an omovertebral bone (green arrow) and associated Sprengel deformity (blue arrow). An unfused cervical spinous process is seen (grey arrow). No IV contrast was administered due to renal disease.

**Fig. 4:**
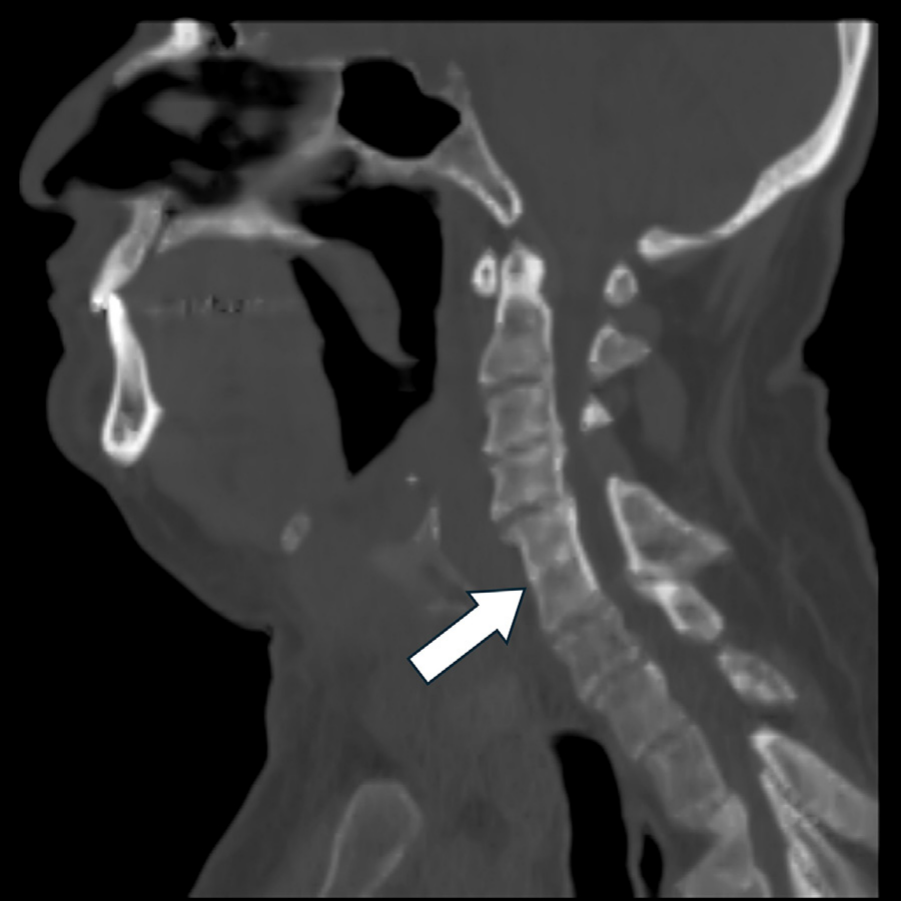
Sagittal CT shows fusion of C5 and C6 vertebrae, including the vertebral bodies (white arrow) and posterior elements. There is narrowing of the anteroposterior dimension of the C5 and C6 vertebral bodies. These findings are consistent with Klippel-Feil syndrome. No spinal hardware is seen to indicate prior surgery.

Chest radiography and the neck CT also provided imaging of bony structures and revealed signs of a Sprengel deformity, including an elevated left scapula and the presence of a left omovertebral bone (Fig. 2, Fig. 3). Additionally, the CT findings included the fusion of the C5 and C6 vertebrae, indicative of Klippel-Feil syndrome (Fig. 4). This fusion was clearly demonstrated on coronal and sagittal reformatted images, allowing for assessment of cervical spine morphology. The imaging findings of congenital cervical vertebral fusion are critical for diagnosing KFS, as they highlight the structural anomalies associated with this syndrome. Thus, CT played a pivotal role in not only characterizing the neck mass but also in identifying the underlying congenital abnormalities.

**Fig. 5:**
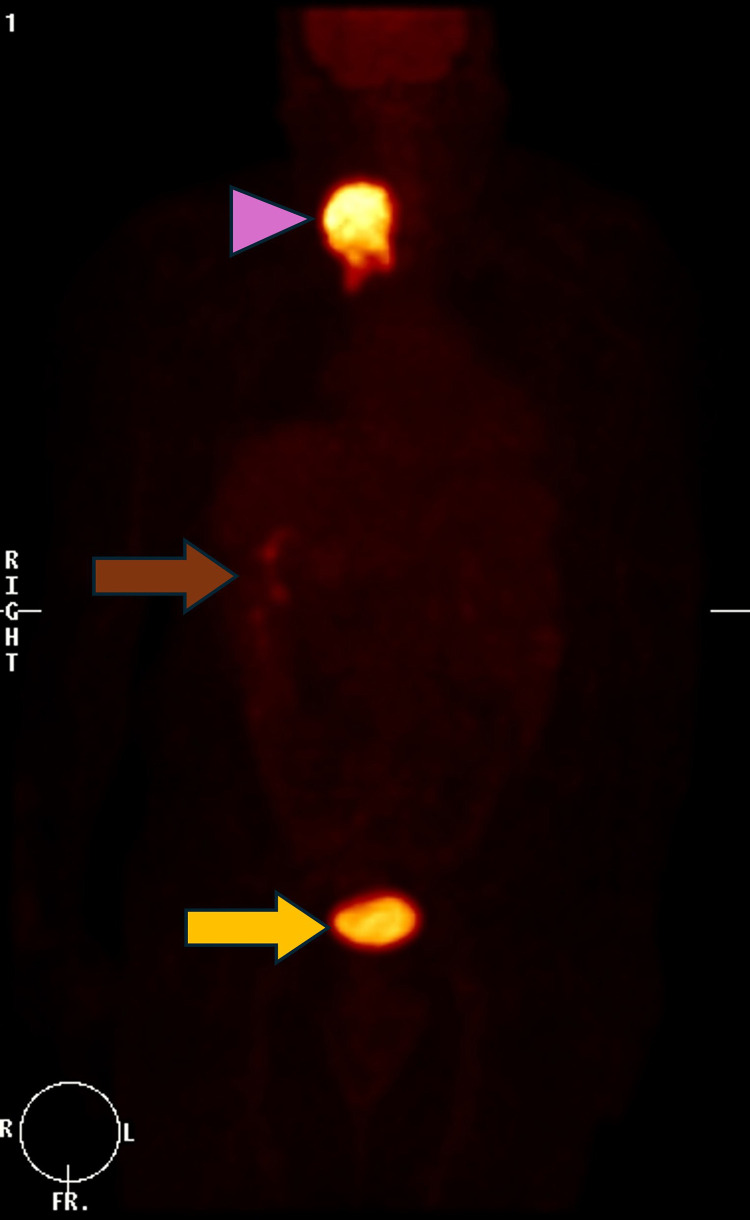
Whole body F-18 FDG PET shows a large area of intense uptake in the right neck (pink arrowhead). There is mild uptake in the region of the gallbladder fossa in this patient with chronic liver disease (brown arrow). Physiologic excretion of radiotracer is seen in the urinary bladder (yellow arrow).

**Fig. 6:**
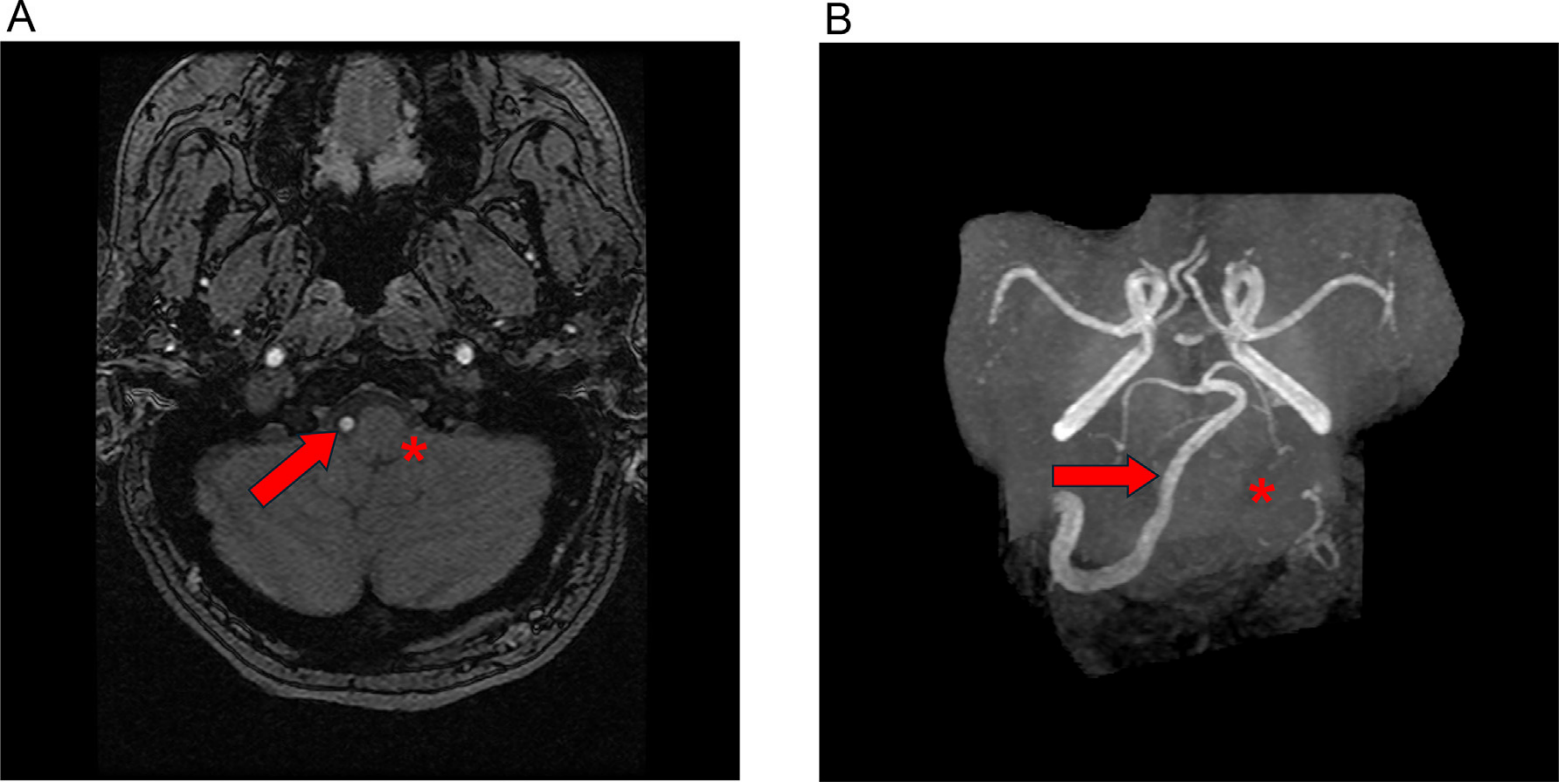
A, B. Head and neck MRI time of flight image and MRA coronal MIP reconstruction show an absent/hypoplastic left vertebral artery (red asterisk) with a normal contralateral right vertebral artery (red arrow).

A whole-body F-18 FDG PET scan demonstrated significant FDG uptake in the right neck mass, with a maximum SUV of 16.7. Mild FDG uptake was also noted in the region of the gallbladder fossa, which correlated with the patient's known liver disease/TIPS and gallbladder wall thickening (Fig. 5). Additionally, the patient's history included an episode of altered mental status. MRI and MRA of the head and neck demonstrated an absent or markedly diminutive left vertebral artery (Fig. 6). While this may be an anatomic variant, it could be related to sequelae of the left omovertebral bone.

Ultrasound-guided biopsy of the right neck mass was consistent with DLBCL. This case represents an exceptionally rare instance of Klippel-Feil syndrome and Sprengel deformity in an adult, complicated by a diagnosis of diffuse large B-cell lymphoma. To the best of our knowledge, there are no other cases of lymphoma reported in the setting of KFS. The congenital abnormalities added to the complexity of the case, and imaging helped accurately diagnose the neck mass that otherwise may have been mistaken for a developmental anomaly on physical exam alone. The location of the neck mass in proximity to key neurovascular structures precluded surgical resection. In addition, the cervical fusion anomalies and Sprengel deformity altered normal anatomic landmarks. The patient began R-CHOP (rituximab, cyclophosphamide, doxorubicin, vincristine, and prednisone) chemotherapy for DLBCL but could only tolerate 2 cycles of chemotherapy because of comorbidities including renal and liver disease. He passed away 5 months after the initial CT workup reported here.

## Discussion

Maurice Klippel and Andre Feil initially described Klippel-Feil syndrome in 1912. It is a rare developmental condition characterized by mono- or multi-segmental fusion of the cervical vertebrae. Because there have been few comprehensive population screening studies, it is anticipated that the true incidence of KFS is higher than the estimated incidence of 1/40,000–1/42,000 in the existing literature [1,2]. While KFS may be autosomal dominant, autosomal recessive, or sporadic, vertebral fusion at C2-3 is known to have an autosomal dominant trait whereas fusion at C5-6 has an autosomal recessive form [3,4]. KFS can be associated with a variety of congenital diseases, including Sprengel deformity [4,5]. KFS manifests with a low posterior hairline and patients often suffer from neck and back pain, reduced mobility, and respiratory issues. Although having a single kidney is not a common finding in KFS, other rare occurrences of KFS with unilateral renal agenesis have been documented. This occurrence is thought to be due to the shared embryological origins and the complex genetic developmental process of the systems involved [6].

In addition to the cervical fusion anomaly, a Sprengel deformity was identified in this patient. Sprengel deformity is a rare congenital anomaly of the shoulder. It is caused by the failure of the caudal migration of the scapula during embryonic development. Patients with Sprengel deformity often suffer from cosmetic deformity and limited mobility of the affected shoulder. KFS is seen in 16%-27% of patients with Sprengel deformity [7]. Sprengel deformity was first described by Eulenberg in 1863; however, years later in 1891, it was Otto Sprengel who explained the pathology and proposed a theory behind the disease's existence. Sprengel deformity is often classified with 4 grades based on severity, with Grade 1 being very mild and not noticeable when dressed, and Grade 4 being severe deformity with the affected shoulder elevated by more than 5 cm [8]. In the case of our patient, the left shoulder was elevated by 5.5 cm and was therefore clinically classified as Grade 4 Sprengel deformity.

In about one-third of patients with Sprengel deformity, osseous or fibrous tissue is seen connecting the lower cervical vertebrae and the medial edge of the scapula which can result in further cosmetic and mobility issues [9]. This tissue known as the omovertebral bar was first described by Willet and Walsham in 1880. The omovertebral bar is usually limited to one side and is thought to play a major role in the malpositioning of the scapula. Willet and Walsham described multiple hypotheses for the bone's etiology, including its potential development originating from the vertebrae, the scapula, or independently as an ossification of the connective tissue between the posterior cervical muscles [9]. Treatment options for Sprengel deformity associated with an omovertebral bar are influenced by several factors, including the degree of shoulder abduction restriction and the symptoms of myelopathy. Surgical management is warranted in severe cases, often to improve the cosmesis and function of the shoulder.

To the best of our knowledge, no association between KFS or Sprengel deformity and DLBCL has been documented in the literature. DLBCL is the most common type of non-Hodgkin lymphoma, accounting for approximately a third of all lymphoma cases worldwide [9]. Patients with DLBCL often present with a rapidly growing mass in single or multiple nodal or extranodal sites. Advancements in our understanding of the immune system's complexity, including B cells, T cells, and natural killer cells, have led to various lymphoma classifications over the past 5 decades. The consensus classification is outlined in the 2016 edition of the World Health Organization (WHO) guidelines [10]. According to these guidelines, diffuse large B-cell lymphomas are characterized as a heterogeneous group of malignancies composed of large cells with nuclei that are at least twice the size of a small lymphocyte and are typically larger than those of tissue macrophages [11,12]. In our patient, DLBCL manifested as a large mass in the right neck without any other sites of involvement.

Multi-modality imaging was critical in our case as the sequalae of KFS can significantly distort normal anatomic landmarks. Imaging was essential in delineating the intricate anatomic relationships of the neck mass, which made surgical resection unfeasible. The patient's comorbid conditions of kidney and liver disease restricted the chemotherapy dosage he could receive and therefore hindered treatment.

Although DLBCL and KFS have not been previously associated, dermoid cysts and malignant teratomas have been reported in association with KFS. Teratomas and lymphomas are distinct entities, but there can be genetic overlap. Shared genetic mutations, such as those in the KIT, RAS, and TP53 genes, might contribute to the development of both tumors [13]. This case highlights the importance of meticulous imaging and clinical evaluation in these conditions, emphasizing the need for awareness of potentially rare associations in patients presenting with congenital abnormalities.

## Conclusion

The concurrent presence of KFS and DLBCL in this case may be coincidental but warrants further investigation. To our knowledge, there are no documented cases in the literature of KFS, Sprengel deformity, and DLBCL occurring simultaneously. This case underscores the importance of comprehensive imaging and clinical evaluation in patients with multiple congenital and acquired abnormalities, as it highlights the possibility of rare associations that may impact diagnosis and management. Multidisciplinary collaboration is essential to navigate the complexities presented by such cases.

## Patient consent

Informed consent for this case was obtained from the patient's next of kin.
